# What exactly is ‘*N*’ in cell culture and animal experiments?

**DOI:** 10.1371/journal.pbio.2005282

**Published:** 2018-04-04

**Authors:** Stanley E. Lazic, Charlie J. Clarke-Williams, Marcus R. Munafò

**Affiliations:** 1 Quantitative Biology, Discovery Sciences, AstraZeneca, Cambridge, United Kingdom; 2 School of Physiology, Pharmacology and Neuroscience, University of Bristol, Bristol, United Kingdom; 3 MRC Integrative Epidemiology Unit, School of Experimental Psychology, University of Bristol, Bristol, United Kingdom

## Abstract

Biologists determine experimental effects by perturbing biological entities or units. When done appropriately, independent replication of the entity–intervention pair contributes to the sample size (*N*) and forms the basis of statistical inference. If the wrong entity–intervention pair is chosen, an experiment cannot address the question of interest. We surveyed a random sample of published animal experiments from 2011 to 2016 where interventions were applied to parents and effects examined in the offspring, as regulatory authorities provide clear guidelines on replication with such designs. We found that only 22% of studies (95% CI = 17%–29%) replicated the correct entity–intervention pair and thus made valid statistical inferences. Nearly half of the studies (46%, 95% CI = 38%–53%) had pseudoreplication while 32% (95% CI = 26%–39%) provided insufficient information to make a judgement. Pseudoreplication artificially inflates the sample size, and thus the evidence for a scientific claim, resulting in false positives. We argue that distinguishing between biological units, experimental units, and observational units clarifies where replication should occur, describe the criteria for genuine replication, and provide concrete examples of in vitro, ex vivo, and in vivo experimental designs.

## Introduction

Designing experiments is challenging: there are many options to consider, decisions to make, and trade-offs to weigh, and a single poor design choice can make an experiment nearly worthless. We focus here on replication—a critical part of experimental design that is often misunderstood [1–7], leading to poor design choices, which in turn contribute to irreproducible or meaningless results. The term ‘replication’ has several related meanings, and here it refers to the classic statistical definition of an intervention or treatment applied to multiple biological entities (experimental units [EUs]) [8–13]. It does not refer to researchers trying to reproduce or replicate their own or others' results.

Both statisticians [8–11,13] and biologists [3,7,14–16] agree on the importance of replication and distinguish between two types. The first is replication that increases the sample size (*N*) and thus contributes to testing an experimental hypothesis. It is called true, genuine, or absolute replication, and when these qualifiers are not used, replication is understood to mean this type. The second type is replication that does not increase the sample size and is called pseudoreplication [3]. Confusing pseudoreplication for genuine replication artificially inflates the sample size, thereby inflating the apparent evidence supporting a scientific claim, and contributes to irreproducible results. An example will illustrate the difference: suppose a researcher hypothesises that male mice have heavier brains than female mice. He could (1) weigh the brain of 1 male and 1 female mouse 5 times, or (2) weigh the brain of 5 male and 5 female mice once. Both designs provide 10 data points to calculate a *p*-value, but the *p*-value is meaningless for the first design because the hypothesis is about sex differences, and there is only 1 member of each sex. The multiple measurements on these 2 mice do not contribute to *N* and thus constitute pseudoreplication. The wrong choice of replicate cannot be fixed by a clever statistical analysis after the experiment is completed (e.g., using multilevel or hierarchical models); it needs to be planned at the design stage. Sometimes it is less clear what aspect of an experiment should be replicated to increase the sample size, and far too often the wrong aspect is chosen [1–7].

Pseudoreplication is not a new problem. Dunn discussed the distinction in 1929 [1], and papers and books warning of the genuine versus pseudoreplication distinction have appeared regularly (with four papers [17–20] and a book chapter [21] in the past year). But, paraphrasing Goodman's comment on misinterpreting *p*-values, ‘these lessons appear to be either unread, ignored, not believed, or forgotten as each new wave of researchers is introduced to […] research’ [22].

What do we hope to achieve with yet another discussion? First, we argue that the frequent and common distinction made between ‘biological’ and ‘technical’ replication is unhelpful because they are inconsistently defined, do not capture the important characteristics of an experiment, and do not clarify what to replicate. We introduce instead the concept of biological units (BUs), EUs, and observational units (OUs), and argue that they can clarify where replication needs to occur [21]. Second, we provide detailed criteria for genuine replication that are applicable to all biological experiments and that researchers can use to design their experiments. We also remark on the assumptions that are made if a criterion is not met. Finally, we provide concrete examples to help translate the general principles and criteria into practice.

## Why is it hard to identify genuine replication?

Pseudoreplication problems arise from the organisational complexity of life itself. Recall the ‘hierarchy of life’ from your first biology class, which starts with atoms at the bottom and ends with the biosphere at the top. Most biomedical research takes place in the middle of the hierarchy, between macromolecules (e.g., DNA, protein complexes) and whole organisms. Multiple levels of biological organisation complicate experiments in two ways. First, the hierarchy imposes a top-down influence, where properties at one level are influenced by those above; for example, the properties or characteristics of an eye (organ) depend on the rat in which it is located (organism). We might expect visual acuity to differ between old and young rats, and it follows that two eyes from the same rat will tend to be more alike than eyes from two different rats. If the rats are the same age, the within-rat variation might be similar to the between-rat variation, but some additional rat-to-rat variation is expected. The same principle applies throughout the hierarchy; two cells in the same eye will tend to be more alike than cells between different eyes (within a rat), and two mitochondria within a cell will tend to be more alike than mitochondria between two different cells. Thus, if we are interested in visual acuity, ‘Rat’ is a variable that predicts or affects visual acuity, before any experimental intervention is applied.

Second, the research hypothesis, the experimental manipulations or interventions, and the measurements, could all operate at different levels, making it unclear which level should be used to determine the sample size—is *N* the number of rats, eyes, cells, or mitochondria? Entities quickly multiply as we go lower in the hierarchy; each rat has two eyes, each eye has many cells, and each cell has many mitochondria. Ideally, the hypothesis, manipulations and/or interventions, and measurements would all be at the same level of biological organisation, but many experiments span multiple levels.

In addition, technical hierarchies can make experiments more complex; for example, an in vitro experiment using a single cell line might be independently replicated on 3 separate days, with wells in a microtitre plate randomised to different treatment conditions, and measurements taken on several cells within each well. Is the sample size the number of days, wells, or cells? Or, does *N* = 1 because only 1 cell line was used?

## What is the extent of the problem?

**Fig 1:**
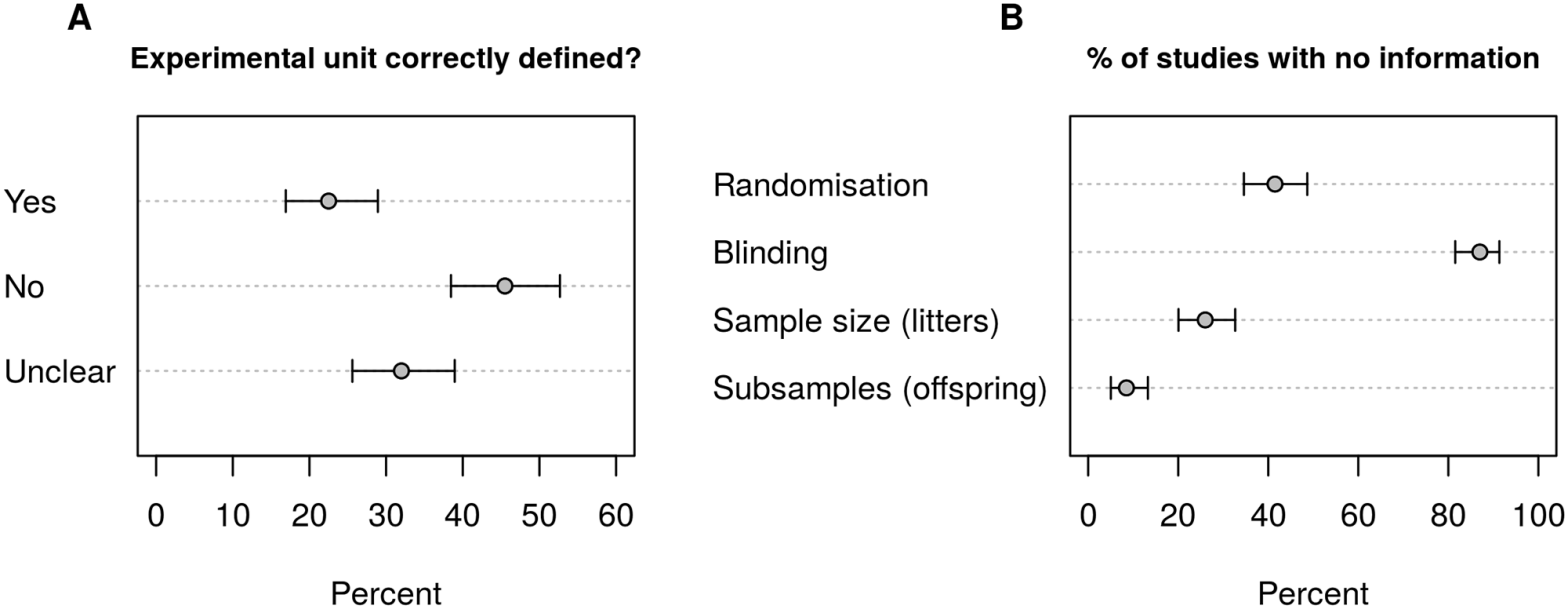
Results of the literature review. Nearly half of the studies (46%, 95% CI = 38%–53%) confused pseudoreplication for genuine replication while 32% (95% CI = 26% to 39%) did not provide enough information to determine if the sample size was correct (A). Consistent with previous research, randomisation, blinding, and the sample size was not always reported (B). Error bars are 95% CI.

The results of the literature survey (Box 1) were disappointing but in line with previous findings [26–28] (Fig 1A). We found that only 22% of studies (95% CI = 17%–29%) had genuine replication and thus made valid statistical inferences. Nearly half of the studies (46%, 95% CI = 38%–53%) had pseudoreplication while 32% (95% CI = 26%–39%) did not provide enough information to determine if *N* corresponds to genuine replication or pseudoreplication. We suspect that most studies in the Unclear category had pseudoreplication because if researchers were aware of this problem, they likely would have described how they avoided it.

**Box 1. How we chose the studies**

We searched PubMed for animal studies where experimental interventions were applied at one level (the parents) but effects were examined at another level (in the offspring). We focused on studies with this design because regulatory authorities provide clear guidelines on replication [23,24]. The following search term was used: "prenatal exposure [TIAB] AND offspring [TIAB] AND ("2011/01"[DP]: "2016/09"[DP]) NOT review [PT]", restricting the search to non-review articles between January 2011 and September 2016. Five-hundred abstracts were returned, and from this list we randomly selected 200 abstracts to examine further. The inclusion criteria were: (1) English language paper; (2) multiparous mammals used in the experiments; and (3) paternal or maternal animals assigned to treatment conditions and the treatment applied to them, but the outcomes measured in the offspring. These criteria excluded studies on humans, nonhuman primates, and nonmammalian species. Papers deemed irrelevant were discarded and another randomly selected paper was included until we reached 200 papers that met the inclusion criteria. We chose 200 papers as that gives a target 95% CI width of approximately 10%. Based on a previous study [25] we expected that 80%–90% of papers would mistake pseudoreplication for genuine replication, which gives a 95% CI width of between 9% and 12% (the CI width depends on the percentage of studies with pseudoreplication).

For each paper, we assessed whether *N* reflects genuine replication or pseudoreplication and classified papers into ‘Yes’, ‘No’, and ‘Unclear’ (insufficient information). Pseudoreplication can be determined when the residual degrees of freedom (df) are greater than the number of parents (the residual df equals the sample size [*N*] minus the number of parameters in the model, and so can never be greater than the number of parents). In addition, we recorded if the paper mentioned using randomisation and blinding, if the number of litters (or equivalently, the number of dams or paternal animals) and the number of offspring were reported, and if the experiment used a split-unit design. In a split-unit (also known as a split-plot) design, randomisation occurs at multiple levels in a hierarchy; for example, pregnant dams (and their unborn offspring) are randomised to treatment conditions, and when the offspring are born, they are randomised to another set of treatment conditions.

Since both blinding and randomisation can occur at several places in an experiment, as long as authors mentioned using them once then we assumed they were used in all appropriate places (even if the methods were not mentioned again elsewhere), and coded the manuscript as having used these methods. Papers with multiple animal experiments were classified as having used blinding or randomisation for all experiments if the authors mentioned these methods for at least 1 experiment. Data are available at the University of Bristol data repository, data.bris, at https://doi.org/10.5523/bris.2uad9gecss2r2ksaujt85gj4a.

Pseudoreplication occurs, strictly speaking, when the statistical analysis is inappropriate for the design of the experiment, usually because the analysis uses the incorrect entity for the EU. Some might argue that a reanalysis with the appropriate model removes all concerns about pseudoreplication. This is not true because the correct analysis can have such low statistical power that the experiment is of little use. For example, if 2 animals per litter are used in the above experiments—the minimal amount of pseudoreplication possible—the appropriate analysis reduces the sample size by half, leading to a large reduction in power. Unfortunately, the number of pseudoreplicates is often greater than 2, and in a field such as neuroscience where the median power of experiments is between 8% and 31% [29], further power reductions are unacceptable. Although pseudoreplication is an analysis problem, it can only be solved when designing an experiment by ensuring replication occurs at the right hierarchical level to provide a reasonable sample size.

Furthermore, 42% of studies (95% CI = 35%–49%) did not report using randomisation and 87% of studies (95% CI = 82%–91%) did not report using blinding (Fig 1B). In addition, 26% of studies (95% CI = 20%–33%) did not report the sample size (number litters/dams/paternal animals) and 8.5% (95% CI = 5%–13%) provided no information on the number of offspring. Half of the studies (48%; 95% CI = 41%–56%) did not report the number of offspring for all experiments, or at least it was unclear how the total number of offspring was divided into subgroups for different experiments or readouts.

## Requirements for genuine replication

To ensure that the results can be extrapolated to the population of interest, there must also be replication relevant to the hypothesis being tested. For this, it helps to define the scientific or BU of interest, which is the entity (i.e., people, animals, and cells) that we would like to test a hypothesis or draw a conclusion about. For example, to conclude that a drug is better than a placebo, a large number of patients are required because the hypothesis is about patients. We cannot give Jim the drug and Bob the placebo, take a daily measurement for several weeks, and then make general conclusions about the drug's efficacy. At times, the BU may not be so apparent. Suppose we hypothesise that outbred mice are smarter than inbred mice. We cannot test this hypothesis with only 1 strain of outbred and 1 strain of inbred mice, even if we have many mice from each strain. There may be intelligence differences between these 2 strains that have nothing to do with their inbred/outbred status, much like there are differences between Bob and Jim that cannot be disentangled from the drug effect. The BU is the strain (because the hypothesis is about strains) and therefore we need multiple strains of both outbred and inbred mice.

Similar problems arise in cell culture studies. If we hypothesise that breast cancer cell lines proliferate at a faster rate than lung cancer cell lines, we need multiple breast and lung cell lines, as the hypothesis is about differences between the tissue of origin. If 1 breast and 1 lung cell line proliferate at different rates, we cannot attribute this to the tissue of origin as no 2 cell lines are expected to proliferate equally.

Having defined the BU in the experiment, the next entity to define is the EU, which is the entity that is randomly and independently assigned to the treatment conditions. It is sometimes called the unit of allocation or the unit of randomisation and examples include a person, animal, litter, cage or holding pen, fish tank, culture dish, or well in a microtitre plate. In many experiments the BU and EU might be the same entity, and the key point, which we return to, is that the sample size corresponds to the number of EUs [11–13,21,30].

Finally, we can define the OU in an experiment (also known as the measurement unit), which is the entity on which measurements are made; for example, are we measuring dendrites (e.g., their length), nuclei (size), hands/paws (grip strength), or people or animals (body weight). Since there are often multiple OUs per EU, pseudoreplication can be introduced if the number of OUs is incorrectly treated as *N* (Box 2). When all three units correspond to the same entity, the design and analysis of an experiment is simpler, but when the units refer to different entities, the intuition behind what constitutes *N* breaks down.

**Box 2. Types of units (adapted from Lazic [21])**

**Biological unit of interest (BU)**: the entity about which inferences are made. The purpose of an experiment is to test a hypothesis, estimate a property, or draw a conclusion about BUs.

**Experimental unit (EU)**: the entity that is randomly and independently assigned to experimental conditions. The sample size (*N*) is equal to the number of EUs. They may correspond to:

1. A BU of interest
2. Groups of BUs
3. Parts of a BU
4. A sequence of observations on a BU

In addition to random and independent assignment, for genuine replication:

1. The treatment(s) should be applied independently to each EU, and
2. The EUs should not influence each other.

If these conditions do not hold, using a different unit as the EU may be preferable, usually a unit one level up in the biological or technical hierarchy.

**Observational unit (OU)**: the entity on which measurements are taken, which may be different from the EUs and BUs of interest. Increasing the number of OUs does not increase the sample size.

**Fig 2:**
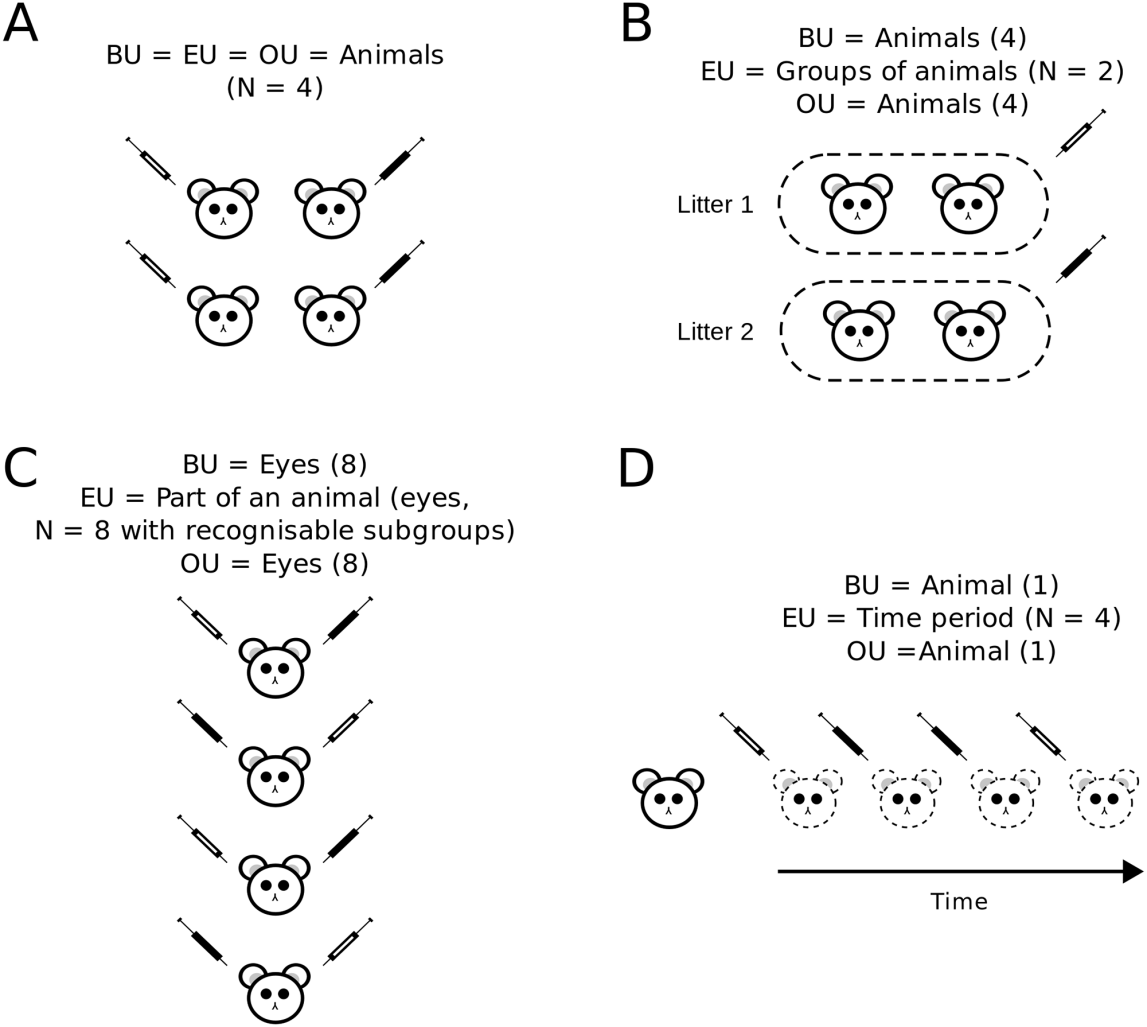
Types of EUs. An EU can correspond to a BU of interest such as an animal (A); groups of BUs, when they are randomised by group or when the treatment is applied group-wise (B); or parts of a BU, where each animal forms the ‘recognisable subgroup’ (C). A less common example is when an experiment is divided into time periods that are randomly assigned to different treatment conditions and a measurement is taken during each time period. Note how the number of animals may differ from the sample size (*N*). The 2 experimental conditions are represented with syringes (white = control, black = treated). Adapted from Lazic [21]. BU, biological unit; EU, experimental unit.

Although the EU often corresponds to a BU of interest (Fig 2A), it can also correspond to a collection or group of BUs, such as all mice in a litter if the treatment is applied to the pregnant dam (Fig 2B). In addition, the EU can correspond to parts of a BU; for example, individual eyes, patches of skin, or organotypic slice cultures from the same animal, as long as these parts can be randomly and independently assigned to different conditions (Fig 2C). Finally, an EU can correspond to a sequence of observations on a single BU. For example, the experiment is divided into time periods that are randomly assigned to different treatment conditions (e.g., on or off a drug), and a measurement is taken at each time period (Fig 2D). This last design is infrequent in biological experiments but forms the basis for *N*-of-1 and crossover designs (and differs from a longitudinal or repeated-measures design). Below we use concrete examples to illustrate these points.

**Fig 3:**
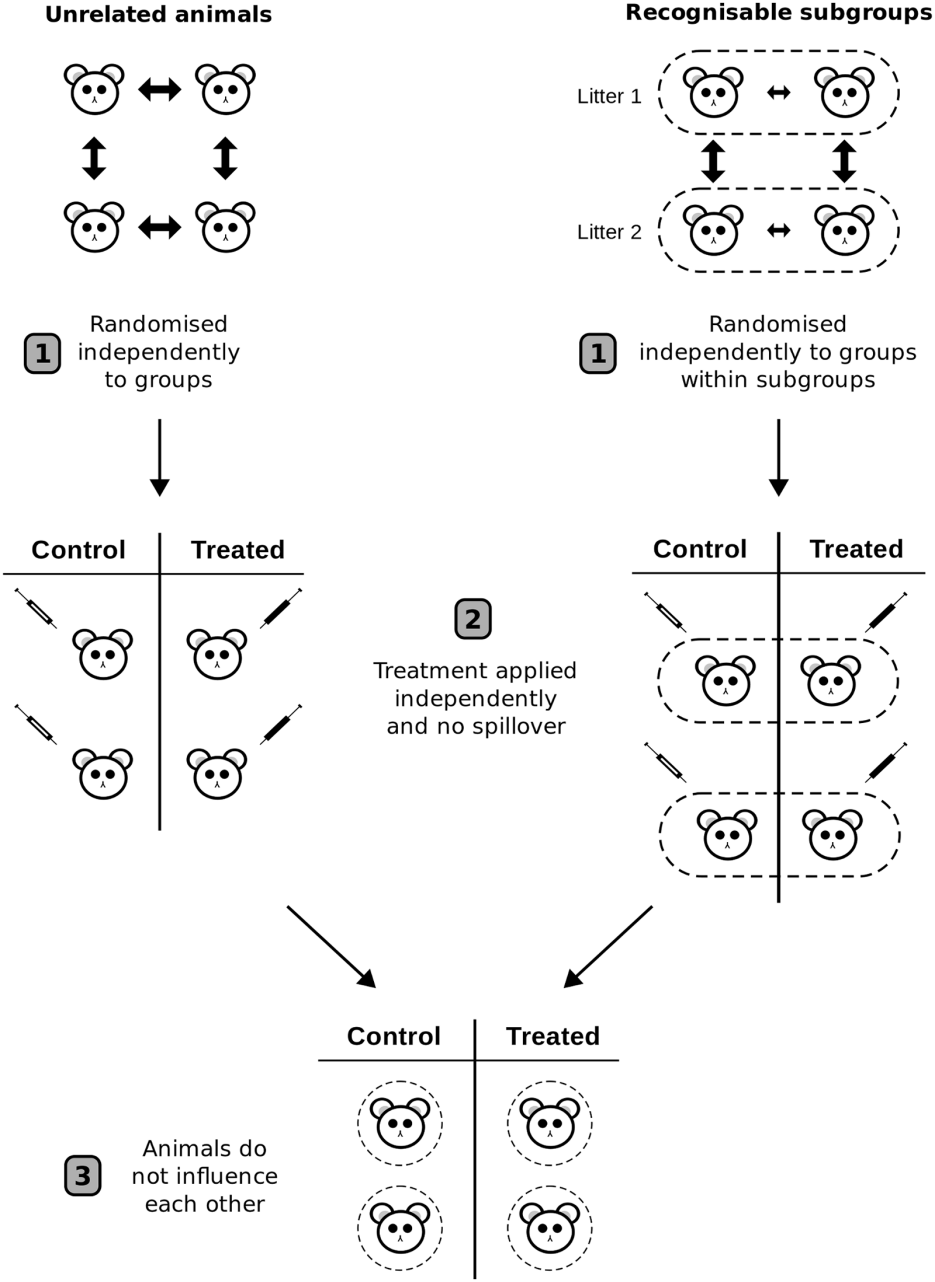
Three criteria for genuine replication. (1) EUs (animals) must be independently randomised to the treatment conditions. Double arrows indicate variability between EUs, and is constant when the EUs are homogeneous. This variability between the EUs is used to test for differences between treatments. If there are recognisable subgroups such as litters or sex, randomisation can be done within each subgroup. Variability will tend to be less between EUs in a subgroup (smaller arrows) than between subgroups. (2) The treatment must be independently applied to each EU and must not affect or spillover to adjacent EUs. The syringes represent the application of an intervention to the EUs (white = control, black = treated). (3) The EUs must not influence each other (dashed circles represent animals isolated from influencing each other), especially on the outcomes of interest. Adapted from Lazic [21]. EU, experimental unit.

As mentioned previously, to increase the sample size we need to increase the number of EUs (a useful phrase to remember is ‘sample size is where you randomise’) [21]. But there are three conditions that must be met for an EU to be considered a genuine replicate (Fig 3). First, EUs must be independently allocated to experimental conditions or interventions. Independence is required because *p*-value calculations assume independence. A *p*-value measures the discrepancy of the observed results to a theoretical null distribution. Since the null distribution is based on hypothetical alternative randomisations of the EUs to experimental conditions, we get a different null distribution (and *p*-values) if potential EUs are randomised independently or in batches or subgroups (e.g., animals in the same litter always end up in the same condition). A related concern is how these subgroups are formed. Are they natural groupings of BUs such as animals in a litter, or all cells in a tissue sample? If so, then we expect the potential EUs within a subgroup to be more alike on relevant variables than EUs between subgroups, as discussed earlier. When the pool of EUs to be randomised have such ‘recognisable subgroups’, which are typically defined by biological factors such as sex, litter, age, and so on, it makes sense to ensure that the subgroups are balanced across the treatment conditions. A balanced design can be achieved by randomising within each subgroup separately (Fig 3, right side).

The second requirement is that the experimental intervention must be applied independently to each EU. Independence is needed here because we cannot exactly replicate the application of a treatment to all EUs—known as treatment error. For example, intraperitoneal injections are never in exactly the same location and the same amount, but when given independently to each rodent, these treatment errors average out across the experimental groups. If we accidentally give a rodent twice the required dose, only this animal will be affected. But if instead we apply the treatment to groups of animals simultaneously, then all animals will be affected the same way (the treatment errors are correlated). For example, if we accidentally give twice the concentration of a drug in the drinking water, then all animals in a cage that share a water bottle will be affected. In many experiments the treatment error will be small compared with the size of the treatment effects we are trying to detect, and barring any experimental mistakes, the treatment error may be negligible. But this is an assumption, and one that is usually untestable. A related point is that the treatments should not spill over or affect adjacent EUs, especially those in another condition. For example, if one side of the brain is infused with a growth factor and the other side serves as a control, we assume that the growth factor does not diffuse to the opposite hemisphere. If we cannot be sure that this assumption holds, and especially if we are unable to verify it, then it may be better to use different animals for the treated and control conditions.

The third requirement is that EUs must not influence each other, especially on the measured outcome variables. For example, Kalbassi recently found that the behaviour of wild-type mice was altered when they were housed with their Neuroligin-3 knock-out littermates (a model of autism) [31]. Here, EUs in one condition are affecting EUs in another. But EUs within the same experimental condition must not influence each other on the relevant outcomes either. Consider a group of students taking a statistics test that are in the same experimental condition and meet all of the above requirements for genuine replication. If the examiner leaves the room and the students collaborate, we expect the variability of the test scores to decrease. The students copy each other’s answers and their responses become more alike (not independent). If we use students as the EU, the variability amongst the test scores is too low, leading to artificially precise estimates, which usually translates into *p*-values that are too small. The mean test score of all students is the only useful piece of information. If weaker students tend to copy the correct answers from stronger students, then the mean test score will be biased, but if only the variability is affected, then the group mean can still be used in an analysis.

We expect animals in a cage to influence each other on many relevant variables, from behaviour to microbiomes (and hence anything influenced by an animal's microbiome). Even if animals meet the first two criteria for genuine replication, mutual influence of animals in the same cage may render them unsuitable to be an EU. The solution is to house animals 1 per cage, or, if this is undesirable for ethical or experimental reasons, housing animals 2 per cage maximises the number of cages (which are now the EUs) for a fixed number of animals. The same idea applies to cells in a well or tissue, if there is mutual influence, or suspected influence, then *N* cannot refer to the number of cells. Next, we show how these ideas apply to different types of experiments.

## Animal experiments

If treatments are randomly and independently applied to an entity other than the individual animal, then the sample size is not the number of animals. The studies in the literature review applied treatments to pregnant females and observed the effects in the offspring. None of the criteria are met for using the offspring as genuine replicates: animals in the same litter are randomised together, the treatment is applied to all animals simultaneously, and it is unreasonable to assume that animals in a womb do not influence each other (e.g., compete for maternal resources). In these experiments the EUs (pregnant dams or occasionally the fathers) do not correspond to the BU of interest (offspring) [25], and regulatory authorities do not allow the offspring to be used as independent samples for toxicology studies using this design [23,24].

What about a similar experiment where, after offspring are born, they are randomised by litter to treatment groups, so that all animals in a litter end up in the same treatment group? Now the treatment can be applied independently to each animal (second criterion) and animals can be prevented from influencing each other (third criterion). But since the animals are not independently randomised (although they could have been), the first criterion is not met and the litter is the EU. This experiment may be less misleading if offspring are treated as the EU since two of the three criteria are met, but litter is still the appropriate EU. Another problem with this design is that the variable ‘litter’ is nested under the variable ‘treatment group’, which is a less powerful design compared with crossing litters and treatment groups [21]. In the crossed arrangement (Fig 3, right side), the individual animals are randomised to the treatment condition, so *N* is the number of animals. This is a powerful design even if there are large differences between litters because the litter-to-litter variation can be cleanly estimated and removed by including litter as a variable in the analysis. A nested arrangement is rarely a good idea because the BU and EU do not coincide, it is harder to analyse, and it is less powerful than the crossed design. The same idea extends to more than 2 animals per litter and additional experimental groups.

In some experiments, unrelated animals may be (1) individually randomised to treatment groups and then housed together by their assigned treatment group or (2) individually randomised to cages and then cages are randomised to treatment groups. In both cases animals, and not cages, can be considered the EU because the animals are independently assigned to the treatment groups (first criterion is met), but the second and third criteria must also be met for animals to be considered the EU.

In other experiments unrelated animals may be randomised to treatment groups and the treatment applied cage-wise in the drinking water or to individual animals by gavage. When a treatment is applied cage-wise, the cages are the EUs because the treatment is applied simultaneously to all animals in a cage (second criterion not met), whereas when the treatment is applied independently to each animal, the animal is the EU. For many experiments the difference between these two designs may be negligible if the treatment error is low, but nevertheless, it is important to know the assumptions made.

Nonhuman primate experiments tend to have small sample sizes for both cost and ethical reasons, but the requirements for genuine replication remain the same. Suppose an experiment records from a single neuron while pictures of a happy monkey or a sad monkey are shown to the subject. One-hundred pictures are shown in random order, and for each trial the firing rate of the neuron is measured. We find that the neuron fires at a faster rate in the happy monkey condition, and the sample size is the 100 trials (this design resembles Fig 2D). This seems to be an easy way of obtaining a large sample size with only 1 subject and only 1 neuron, but the catch is that the results apply only to this subject and to this neuron, and little can be said about what might be seen in other subjects or other neurons. A statistical test would be valid, but the hypothesis tested is uninteresting. One may argue that this subject is representative of others, but this is a nonstatistical generalisation, and the smallness of the *p*-value does not provide more evidence about what might happen in other subjects (showing that a drug works for Jim (*p* < 0.001) does not provide strong evidence that it works for Bob, or anyone else).

Suppose this experiment includes a second subject, run under identical conditions, with similar results. There are now 200 data points, 100 to test for an effect within each subject, but still only *N* = 2 to test for an affect across subjects (showing that a drug works for Jim [*p* < 0.001] and that it also works for Bob [*p* < 0.00001] provides little evidence about what might be seen in others). There are two hypotheses here, which must be reflected in the analysis and interpretation. Strong conclusions cannot be made about subjects in general from these two subjects.

In another variation, suppose that we have only 1 subject, but can record from 10 neurons simultaneously. *N* is not increased 10-fold because showing a picture is the application of the treatment, and it is applied to the subject and to all neurons simultaneously (second criterion is not met, much like applying a drug to all the cells in a well). Furthermore, we cannot rule out that the neurons are not influencing each other—they may be connected with gap junctions or have synaptic connections for example (third criterion is not met).

## Slice preparations and histological samples

In some experiments, animals are randomised to treatment conditions and an intervention is applied to the animals. Then, an organ or body part is examined, usually postmortem. The EU is the animal and the sample size (*N*) is the number of animals. Multiple histological sections, neurons per section, spines per neuron, and so on, are all subsamples as they have been randomised together (first criterion is not met), the treatment is applied simultaneously (second criterion is not met), and they may influence each other between the treatment application and when the tissue is fixed (third criterion is often not met).

In other experiments, a body part is first removed from the animal, the treatment applied to the body part, and then observations are made on the body part. If there are multiple body parts per animal such as the left and right hippocampus, two kidneys, or several blood samples, each of these can be randomised to different treatment conditions. The situation is identical to the right side of Fig 3, but instead of litters, the subgroups are the animals and the body parts are randomised to treatment groups within animals. Here, *N* is the number of body parts, but it is still useful to have multiple animals as this can establish the robustness of the effect, allow one to assess how the effect varies across animals, and makes generalisation to other animals possible.

Some ex vivo experiments are similar to the nonhuman primate example above, where, for example, an organotypic hippocampal culture has time periods randomised to the presence or absence of a serotonin receptor antagonist, and at each period electrophysiological recordings are made. Here we substitute the hippocampal culture for the subject, and the presence or absence of the antagonist for the pictures, and all previous points apply. In other ex vivo experiments, the treatment is applied to the animal, and multiple slice preparations are derived from each animal. Here, *N* is the number of animals, not the number of slice cultures.

## Cell culture experiments

In cell culture experiments cells are often both the OUs and BUs of interest, but rarely the EU. Suppose an aliquot of cells is thawed and the cell suspension is pipetted into different wells of a microtitre plate. Cells are randomised to wells, and then wells to treatments, so the first criterion is met. But treatments are applied simultaneously to all cells in a well, not independently to each cell, so the second criterion is not met. In addition, it is unreasonable to assume that cells in a well have no influence on each other; they form cell-to-cell connections, release signalling molecules, and compete for the same nutrients in the media. Hence, the third criterion is not met for using cells as the EU. Thus, a well, culture dish, or another plastic container is the appropriate EU for cell culture experiments.

But in vitro experiments are often finicky; the results depend on the unique conditions that vary each time the experiment is run. The experimental material (e.g., a cell line) is often artificially homogeneous and the conditions under which the experiment is run are so narrowly defined that it is hard to know what will happen if the experiment is run a second time. For this reason, in vitro experiments are usually repeated on multiple days, and the number of wells, aliquots, or culture dishes within a day are treated as subsamples. The aim is to establish that the phenomenon is robust enough to survive multiple replications of the entire experimental run or protocol in a highly artificial system. This situation has parallels to the nonhuman primate example above. We could do the experiment on 1 day and use the wells as the EU, and have a large sample size, but then we cannot comment on the generalisability of the results. If the experimental system is sensitive to the many details of how it is carried out, then repeating the whole procedure on multiple days provides further information in a way that using more wells on a single day does not. It provides an estimate of the consistency of the effects across the different experimental runs (days). The multiple wells on each day are then treated as subsamples and do not contribute to *N* (for example, by averaging values across wells in the same condition on each day). This is a scientific judgement about the relevant unit that we would like to make inferences about, and although opinions may differ, using more stringent criteria makes the results more believable.

## Analysis of hierarchical data

The analysis of hierarchical data is a large topic and beyond the scope of this paper, but a simple solution to the problem of pseudoreplication is to reduce the data to a single value for each EU. Reducing the data by calculating the mean of multiple measurements on each EU will often be appropriate, but other numeric summaries such as the slope or area under the curve may capture a feature of interest better [32]. These summary-measure or derived-variable analyses allow standard statistical tests to be used, and will often give the same answer as more complex hierarchical models (see S1 Text for several analyses of an example data set) [33–36].

## Conclusion

There are few ways to conduct an experiment well, but many ways to conduct it poorly. Without identifying the correct EU and having replication in the right place, experiments will likely be of little value or will test an uninteresting hypothesis. Other aspects of experimental design and how to analyse data from the above examples are beyond the scope of this paper, but are discussed in detail elsewhere [21]. We hope this presentation provides researchers with the necessary principles to get the most value out of their experiments.

## Supporting information

S1 Text Six analyses of an example dataset showing the differences between an incorrect analysis and several correct approaches. (PDF)

## Abbreviations

BU: biological unit
EU: experimental unit
OU: observational unit
