# Supplementary material for: What exactly is ‘N’ in cell culture and animal experiments?
Source: PLoS Biol. 2018 Apr 4;16(4):e2005282. doi: 10.1371/journal.pbio.2005282 (PMC5902037; doi:10.1371/journal.pbio.2005282)
Supplement: S1 Text — (PDF) [file pbio.2005282.s001.pdf]

What exactly is ‘N’ in cell culture and animal experiments? Analysis of an example data set

Stanley E. Lazic, Charlie J. Clarke-Williams, Marcus R. Munafo

We compare the results of six models on a data set where pregnant dams were randomised to treatment groups but observations were made on the offspring.

Load packages and set options

```
library(nlme)
library(lattice)
suppressMessages(library(dplyr))
suppressMessages(library(rstanarm))
options(mc.cores = parallel::detectCores())
```

This example uses the VPA data set from the `labstats` R package. In this experiment, six pregnant female mice were randomly assigned to receive an injection of valproic acid (VPA, n = 3) or saline (n = 3) and the outcome variable was the amount of locomotor activity in the offspring. Since the measurements are taken on the offspring, they are the observational units (OU). The research hypothesis is whether prenatal exposure to VPA affects locomotor activity in the offspring, and so the offspring are also the biological units (BU). But since the dams (litters) were randomised to the treatment conditions, the experimental units (EU) are the dams and sample size is therefore the number of dams ($N = 6$). A subset of the full data set is used here to simplify the analysis and so one experimental condition was dropped and only two offspring from each litter are used. The data are originally from Mehta et al. [1].

```
data("VPA", package="labstats")


d <- filter(VPA, drug == "SAL") %>%  # select only SAL drug group
    mutate(act = activity/10000) %>% # put on nice scale for plotting
    select(litter, group, act) %>%   # select key variables
    arrange(litter)                  # order by litter

# the data used for this example
d
```

```
   litter group    act
1       C   SAL 6.7752
2       C   SAL 4.9948
3       D   SAL 6.5769
4       D   SAL 8.1351
5       E   SAL 5.8470
6       E   SAL 5.9803
7       H   VPA 4.5258
8       H   VPA 5.3587
9       I   VPA 5.4880
10      I   VPA 5.3566
11      J   VPA 5.6953
12      J   VPA 5.0725
```

The plot below shows the activity by litter.

```
par(las=1)
beeswarm::beeswarm(act ~ litter, data=d, ylim=c(2,10),
                   pwpch=as.numeric(group), pwcol=as.numeric(group),
                   ylab="Activity (x10000)",
                   xlab="Litter")
legend("topright", pch=1:2, col=c("black","red"), legend=c("SAL","VPA"))
```

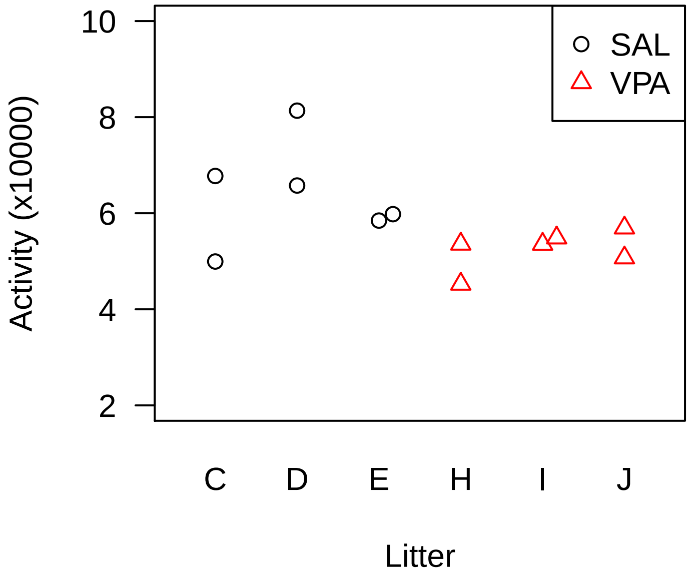

Analysis 1: Pseudoreplication when treating offspring as the EUs

The analysis below analyses all 12 observations, not taking into account that two animals come from each litter and so the number of OUs is twice the number of EUs. Hence, the analysis has an artificially inflated sample size and p-values that are too small.

```
m1 <- aov(act ~ group, data=d)
summary(m1)
```

```
            Df Sum Sq Mean Sq F value Pr(>F)
group        1   3.87    3.87    5.98  0.035
Residuals   10   6.47    0.65
```

The p-value is less than 0.05 and we would conclude that VPA has an effect, but residual degrees of freedom (df) are 10 (shown in the `Df` column). Since the residual df cannot be larger than the sample size (the number of EUs), it is obvious that the analysis is inappropriate.

Analysis 2: No pseudoreplication, the correct EU is specified with "Error()"

The analysis below uses the `Error()` argument to specify that the litters are the EUs, and litter is treated as a fixed effect in this analysis.

```r
m2 <- aov(act ~ group + Error(litter), data=d)
summary(m2)
```

```
Error: litter
          Df Sum Sq Mean Sq F value Pr(>F)
group      1   3.87    3.87    4.97   0.09
Residuals  4   3.11    0.78

Error: Within
          Df Sum Sq Mean Sq F value Pr(>F)
Residuals  6   3.36    0.56
```

There are two tables in the above output `Error: litter` and `Error: Within`. The group variable is found in the `Error: litter` table and has a p-value of 0.09. Unlike the above analysis we would not conclude that there was an effect of VPA (assuming we are making a hard cutoff at $p = 0.05$). Note that the residual df in the `Error: litter` is less than the number of EUs, indicating that there is no pseudoreplication.

Analysis 3: No pseudoreplication, treating litter as a random effect

Another way to specify that the litters are the EUs is to treat litter as a random effect. When the data are balanced, meaning that each litter has the same number of observations, this analysis is equivalent to model `m2`.

```r
m3 <- lme(act ~ group, random=~1|litter, data=d)
anova(m3)
```

```
            numDF denDF F-value p-value
(Intercept)     1     6 521.500  <.0001
group           1     4   4.967  0.0897
```

The p-value is the same as model `m2` (slight differences are due to rounding), and the residual df is also 4 (shown in the `denDF` column).

Analysis 4: No pseudoreplication, average multiple observations on each EU

The simplest way to analyse such data is to reduce the data so that there is only one value per EU. Here, the average of the two offspring in each litter is calculated. This approach is called a *summary measure* or *derived variable* analysis.

```r
# Reduce data by calculating litter average
d2 <- group_by(d, litter) %>%
    summarise(group=unique(group), act = mean(act)) %>%
    as.data.frame()

d2
```

```
  litter group     act
1      C   SAL 5.88500
2      D   SAL 7.35600
3      E   SAL 5.91365
4      H   VPA 4.94225
5      I   VPA 5.42230
6      J   VPA 5.38390
```

```
par(las=1)
beeswarm::beeswarm(act ~ group, data=d2, ylim=c(2,10),
                   pwpch=as.numeric(group), pwcol=as.numeric(group),
                   ylab="Activity (x10000)", xlab="Group")
```

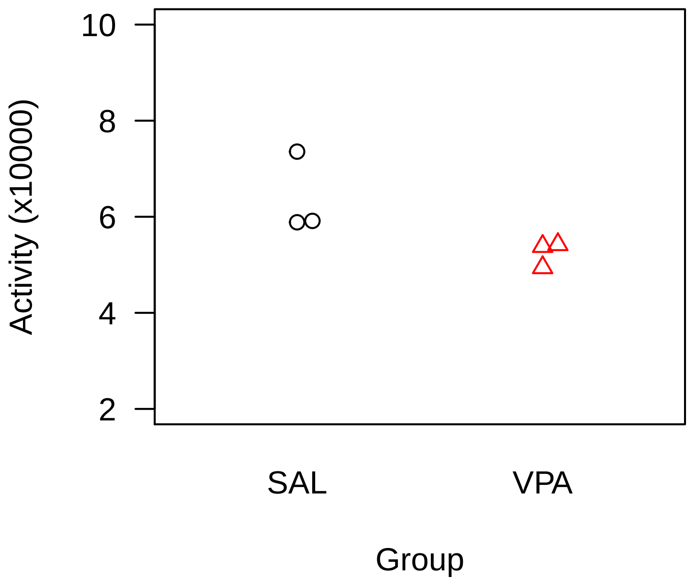

The analysis is now equivalent to a t-test, but using the `aov()` function makes the output consistent with the previous analyses.

```
m4 <- aov(act ~ group, data=d2)
summary(m4)
```

```
            Df Sum Sq Mean Sq F value Pr(>F)
group        1   1.93   1.934    4.97   0.09
Residuals    4   1.56   0.389
```

Once again, we get the same p-value (0.09) and the correct dfs. Averaging multiple observations on each EU won't always give the same results as a mixed effects model (model `m3`), but it occurred here because the number of observations was the same for each EU.

Analysis 5: Bayesian analysis with pseudoreplication

The question of "what is N?" is relevant to both classical frequentist statistics as we showed above, but also to Bayesian methods (Bayesian methods are not discussed here but Kruschke [2] and McElreath [3] provide excellent introductions). Below we fit a model by incorrectly treating all 12 offspring as the EUs; this is the Bayesian equivalent of model `m1`. The priors are chosen to be uninformative and so have little influence on the results.

```r
m5 <- stan_glm(act ~ group, data=d, adapt_delta=0.99, iter=20000,
               prior = normal(0, 20), prior_intercept = normal(5.82, 20),
               prior_aux = normal(0, 20))

summary(m5, pars="groupVPA", digits=3)
```

```
Model Info:

 function:  stan_glm
 family:    gaussian [identity]
 formula:   act ~ group
 algorithm: sampling
 priors:    see help('prior_summary')
 sample:    40000 (posterior sample size)
 num obs:   12

Estimates:
           mean   sd     2.5%   25%    50%    75%    97.5%
groupVPA -1.141  0.549 -2.243 -1.482 -1.137 -0.799 -0.045

Diagnostics:
         mcse  Rhat  n_eff
groupVPA 0.004 1.000 21073

For each parameter, mcse is Monte Carlo standard error, n_eff is a crude measure of effective sample si
```

The above output shows (under the `Estimates` table) that the mean difference between the two groups is -1.141 units, with a 95% CI from -2.243 to -0.045. Since the 95% CI does not include zero, we can (incorrectly) conclude that there is an effect of VPA.

Analysis 6: Bayesian analysis without pseudoreplication

The summary measure analysis below uses the reduced data set, and the same priors as model `m5`. This analysis is the Bayesian equivalent to model `m4`.

```r
m6 <- stan_glm(act ~ group, data=d2, adapt_delta=0.99, iter=20000,
               prior = normal(0, 20), prior_intercept = normal(5.82, 20),
               prior_aux = normal(0, 20))

summary(m6, pars="groupVPA", digits=3)
```

```
Model Info:

 function:  stan_glm
 family:    gaussian [identity]
 formula:   act ~ group
 algorithm: sampling
 priors:    see help('prior_summary')
 sample:    40000 (posterior sample size)
```

```
 num obs:   6

Estimates:
           mean   sd     2.5%   25%    50%    75%    97.5%
groupVPA -1.131  0.950 -2.999 -1.589 -1.133 -0.676  0.763

Diagnostics:
         mcse  Rhat  n_eff
groupVPA 0.008 1.000 14525

For each parameter, mcse is Monte Carlo standard error, n_eff is a crude measure of effective sample si
```

The mean difference is similar to model `m5`, but the 95% CI is now from -2.999 to 0.763. Since zero is included in the interval, we would conclude that VPA has no effect (although Bayesians are unlikely to make hard conclusions about effects being present or absent based on a fixed threshold). To better appreciate the differences in the models, the posterior distributions are plotted below. The posterior distributions represent the uncertainty in the effect of VPA, given the data. Model `m5`, which incorrectly uses a sample size of 12, has an artificially narrower posterior distribution.

```r
# extract draws from the posterior
post_m5 <- data.frame(m5)
post_m6 <- data.frame(m6)

par(mar=c(5,1,1,1))
plot(density(post_m5$groupVPA), xlim=c(-6.1, 4), main="", yaxt="n",
     xlab="Difference between groups", ylab="")
lines(density(post_m6$groupVPA), lty=2, lwd=2, col="grey40")
abline(v=0, lty=2)

legend("topleft", lty=c(1,2), lwd=c(1,2), col=c("black","grey40"),
       legend=c("m5 (N=12)", "m6 (N=6)"))
```

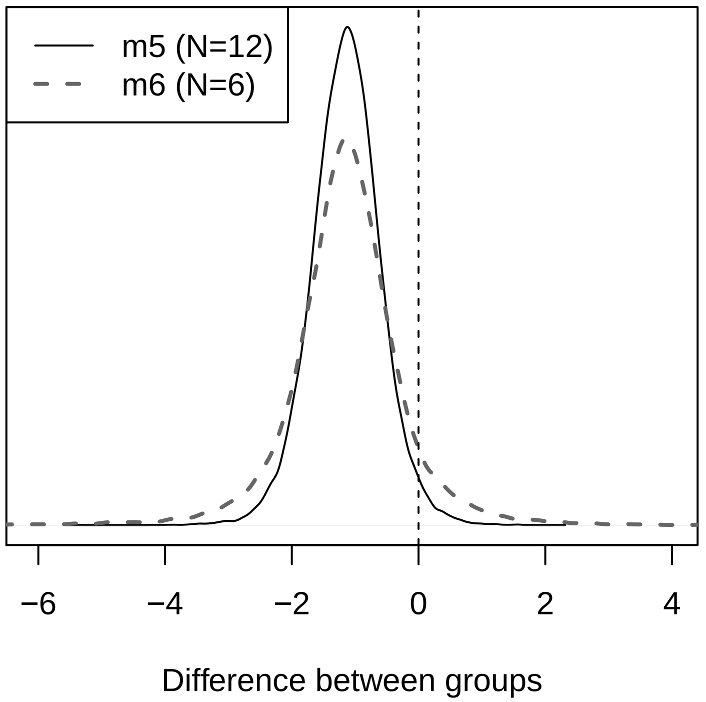

References:

1. Mehta MV, Gandal MJ, Siegel SJ (2011). mGluR5-antagonist mediated reversal of elevated stereotyped, repetitive behaviors in the VPA model of autism. *PLoS ONE* 6(10):e26077.

2. Kruschke JK (2011). *Doing Bayesian Data Analysis: A Tutorial with R and BUGS*. Academic Press, New York, NY.

3. McElreath, R. *Statistical Rethinking: A Bayesian Course with Examples in R and Stan.* CRC Press, Boca Raton, FL.
